# Infrared Thermography Correlates with Lactate Concentration in Blood during Race Training in Horses

**DOI:** 10.3390/ani10112072

**Published:** 2020-11-09

**Authors:** Olga Witkowska-Piłaszewicz, Małgorzata Maśko, Małgorzata Domino, Anna Winnicka

**Affiliations:** 1 Department of Pathology and Veterinary Diagnostics, Institute of Veterinary Medicine, Warsaw University of Life Science, 02-787 Warsaw, Poland; anna_winnicka@sggw.edu.pl; 2 Department of Animal Breeding, Institute of Animal Science, Warsaw University of Life Sciences, 02-786 Warsaw, Poland; malgorzata_masko@sggw.edu.pl; 3 Department of Large Animal Diseases and Clinic, Veterinary Research Centre and Center for Biomedical Research, Institute of Veterinary Medicine, Warsaw University of Life Sciences, 00-797 Warsaw, Poland; malgorzata_domino@sggw.edu.pl

**Keywords:** LAC, exercise, thoroughbreds, sport, muscle metabolism, IRT, sport monitoring

## Abstract

**Simple Summary:**

Thoroughbreds commence race training at a very young age, carrying a high risk for the occurrence of musculoskeletal lesions. Despite clinical examination and trainers’ opinions, the most acceptable way for the accurate and objective evaluation of adaptation to increased exertion is via blood testing. However, this requires blood sampling at three different time points, which may be expensive and stressful for the horse. Additionally, legal regulations often forbid any invasive procedures during equestrian sporting events. As muscle activity increases, there is a progressive increase in body surface temperature. These changes in body surface temperature can be measured by infrared thermography (IRT), which was recently widely incorporated into equine veterinary medicine. However, there is a lack of studies about monitoring race horse training. Thus, the aim of this study was to find a relationship between lactate blood concentration and body surface temperatures, as measured by IRT. This study is the first to present that IRT may supplement blood measurements. In the future, IRT may become an alternative procedure to evaluate horse fitness during race training.

**Abstract:**

In horse racing the most acceptable way to objectively evaluate adaptation to increased exertion is to measure lactate blood concentration. However, this may be stressful for the horse, therefore, a simple, noninvasive procedure to monitor race progress is desirable. Forty Thoroughbreds attended race training, with blood samples collected at rest, immediately after, and 30 min after exercise. The lactate concentration was determined 60 s after blood collection using an Accusport^®^. Thermal imaging of the neck and trunk areas was performed following international veterinary standards from a distance of approximately 2 m from the horse using the same protocol as the blood sampling. The Spearman rank correlation coefficients (ρ) between the changes in the blood lactate concentration and surface temperature measures were found for the regions of interest. The highest positive correlation coefficients were found in the *musculus trapezius pars thoracica* region for the maximal temperature (T Max; ρ = 0.83; *p* < 0.0001), the minimal temperature (T Min; ρ = 0.83; *p* < 0.0001), and the average temperature (T Aver; ρ = 0.85; *p* < 0.0001) 30 min after the exercise. The results showed that infrared thermography may supplement blood measurements to evaluate adaptation to increased workload during race training, however, more research and references values are needed.

## 1. Introduction

The adaptational reaction during race training involves many systems. Thus, an optimal training program is crucial to obtain the best sport results as well as to maintain horse welfare. The training program in very young race horses (starting as yearlings) carries a high risk for the occurrence of musculoskeletal lesions [1]. To prevent injuries despite clinical examination and the trainers’ opinions, exercise tests are performed. Like human athletes, many different protocols of exercise tests are performed and variables are measured in order to calculate the fitness levels of horses. However, an ideal test does not exist, especially in horses [2,3,4]. Performance ability may be expressed by parameters such as speed at a heart rate of 200 beats per minute (V200) and maximum oxygen uptake (VO_2_max). One of the most popular blood biochemical measurements in sports practice is lactate concentration (LAC), which is the most objective evaluation of adaptation to increased exertion [5]. Obtained results may be used to calculate the speed at a blood lactic acid level of 4 mmol/L (VLa4). In superior performance race horses, VLa4 values are high [3]. In addition, decreased LAC accumulation for the same workload indicates increased aerobic capacity [6]. All the above-mentioned tests lead to monitoring of the aerobic capacity. Minimal research was performed to describe the anaerobic power, which may be particularly useful in horses racing over short distances. Higher LAC levels were shown to correlate strongly with increased anaerobic capacity [3,4,7,8,9]. It was documented that the highest plasma LAC was found in the fastest horses on the first 700 m of gallop, but over longer distances the low-LAC-producing horses equaled or beat the high-LAC-producers in terms of speed [10].

In healthy horses at rest, blood LAC values are close to 1 mmol/L [11], which increases during exercise at speeds greater than about 8–10 m/s [10]. LAC accumulation leads to muscular fatigue [12]. However, recent studies show that LAC is also a very important source of energy and mediates many exercise-induced adaptations [13]. LAC removal from the blood depends on the type of training, the intensity and duration, and the fitness of the horse [5,10,14]. Thus, blood lactate monitoring is essential to establish optimum training intensity and adaptation to increasing workload. However, disadvantages of LAC measurements also exist. Standard exercise testing requires blood sampling, which may be stressful for the horse and is therefore not always performed [4,5]. Additionally, law regulations often forbid any invasive procedures during equestrian sporting events. Therefore, a simple, noninvasive procedure allowing assessment of LAC is desirable.

As muscle activity increases, there is a progressive increase in muscle temperature, which leads to an increase in body surface temperature as well. In humans, it was postulated that changes in blood LAC are parallel to an increase in body surface temperature during physical effort [15]. Changes in the temperature of body surfaces can be measured by infrared thermography (IRT) [16]. Recently, IRT was widely incorporated into equine veterinary medicine to detect lameness [17], as well as to estimate the stress reaction during endurance exercise [18], predisposition to effort [19], and to detect performance-enhancing techniques [20]. Correlations between changes in body surface temperature and blood parameters were also investigated in order to identify the predictive value of surface temperature measurements as a marker of animal performance [21]. In this promising research, measurement of hematological parameters, including hematocrit (HCT), and biochemical parameters, such as creatine phosphokinase activity (CPK) and concentrations of glucose (GLU), urea, sodium (Na^+^), potassium (K^+^), and calcium (Ca^2+^), were taken into consideration. However, none of these studies recorded thermal images in connection with changes in LAC during exercise, which is the best parameter for monitoring of training progress.

As a noninvasive procedure, IRT may give additional information on race training. Moreover, it could be used as an alternative procedure to blood measurements. During competition, the ability to obtain measurements from distance is particularly important due to restrictions created by jockey clubs or other equestrian associations, such as Fédération Equestre Internationale (FEI). Thus, the aim of this study was to find a relationship between LAC and body surface temperatures, as measured by IRT during race training.

## 2. Materials and Methods

### 2.1. Animals

The study involved 40 healthy, privately-owned, 2–4-year-old, racing Thoroughbred horses (20 mares and 20 stallions). The horses were kept and trained at the same racing track in similar conditions and trained by one trainer. They were fed with a standard diet designed for race horses (oats 5.5 kg/horse, meadow hay 7.5 kg/horse, and special concentrate for performance horses). According to the owners, all horses were dewormed and vaccinated at similar times and did not receive medications or suffer from infection in the preceding 3 weeks. Basic clinical examinations, including heart rate, mucous membranes (color and moisture), capillary refill time, dehydration (measured as the time it takes for a pinched skin fold over the point of the shoulder to flatten), and rectal temperature, were performed by the veterinary practitioner before and after each training and revealed no clinical symptoms of disease. The training session was performed on the same day to avoid weather influence, at an air temperature 20.2 ± 1.1 °C and a humidity of 45 ± 0.8% [22,23]. The training started with 10 min walk, then 15 min of trotting, followed by 5 min of canter. At the end, a gallop was performed on sand for 800 m at a speed of about 800 m/min for all horses.

### 2.2. Blood Sampling

Blood samples were collected at rest, in the morning, before feeding (measurement 0; LAC 0), immediately after (1–3 min; measurement 1; LAC 1), and 30 min after exercise (measurement 2; LAC 2). All samplings were a part of standard veterinary diagnostic procedures according to Polish legal regulations (art 1.2 (5) Ust. z dnia 15 stycznia 2015 r. o ochronie zwierzat wykorzystywanych do celów naukowych lub edukacyjnych, Dz.U.2018.0.1207 (Resolution on the animals protection used for scientific and educational purposes); the European directive EU/2010/63 approval of the Local Commission for Ethics in Animal Experiments was not required. Blood samples were acquired by a jugular venipuncture using BD Vacutainer^®^ dry tubes (Plymouth, UK). Blood lactate concentrations were determined immediately after blood collection using an Accusport® (Roche Diagnostics, Basel, Switzerland) (LAC, mmol/L).

### 2.3. Thermography

**Figure 1:**
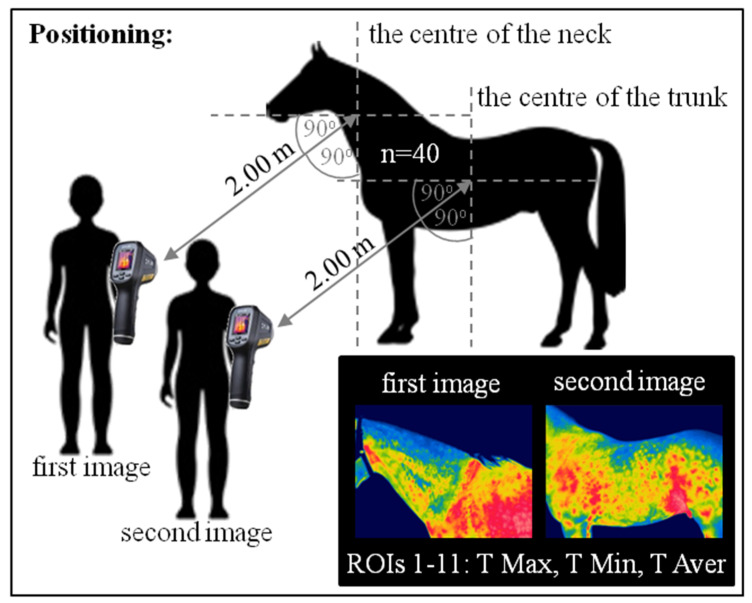
The procedure of the thermographic data collection. The measurements were repeated three times: Before exercise, immediately after exercise, and 30 min after exercise. Two images were taken each time. In each image, 11 regions of interest (ROIs) were determined. The maximal temperature (T Max), the minimal temperature (T Min), and the average temperature (T Aver) were calculated for each of the ROIs.

Thermal imaging was performed following international veterinary standards [24]. The study was performed in late May. A total of 240 images were taken in a closed stable protected from wind and sun radiation to minimize the influence of external environmental conditions [25]. The thermographic images were taken on the left side at a 90° camera angle from a distance of approximately 2 m from the horse using the same protocol as the blood sampling. During each of the three measurements, two images were taken. The first image was positioned on the center of the neck, whereas the second was on the center of the trunk. Images were taken using an infrared radiation camera (FLIR Therma CAM E25, Sorocaba, Brazil), with an emissivity (e) of 0.99 and a temperature range of 20–40 °C. The procedure of the thermographic data collection is presented in Figure 1.

### 2.4. Data Analysis

**Figure 2:**
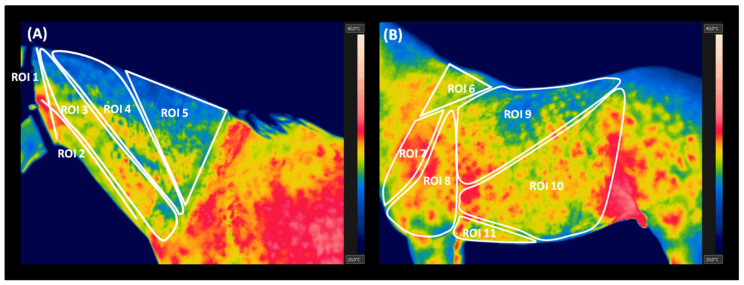
The regions of interest (ROIs) chosen for statistical analysis of the thermographic data. (**A**) The neck area (ROIs of 1–5); (**B**) the trunk area (ROIs 6–11).

Surface temperatures of 11 regions of interest (ROIs 1–11) were evaluated according to the criteria described in Table 1 and Figure 2. The maximal temperature (T Max), the minimal temperature (T Min), and the average temperature (T Aver) of each ROI were calculated using professional software (FLIR Tools Professional). T max and T min represented the values of the highest and the lowest temperatures recorded in consecutive ROIs, respectively, whereas T Aver reported the value of the mean temperature calculated for the entire ROI area. Obtained data were presented in the form of a data series, in which subsequent horses were represented by other realization.

LAC data series and thermal measurements were tested independently for univariate marginal distributions using a univariate Kolmogorov–Smirnov test. Non-Gaussian distribution was stated for following data: LAC 0; LAC 1; LAC 2; T Min 0 in ROI 3; T Aver 0 in ROIs 1, 3 and 5; T Max 1 in ROI 10; T Min 1 in ROIs 1, 3, 4, 7, 8, 10; T Aver 1 in ROI 5; T Max 2 in ROI 4; T Min 2 in ROIs 1–5 and 11; T Aver 2 in ROIs 1 and 3. Other data series showed normal distribution. Temperature data series were compared as paired data to estimate the differences between measurements (0, 1, 2) and between ROIs within each measurement using a repeated measures ANOVA summary with Tukey’s multiple comparisons test or Friedman test with Dunn’s multiple comparisons test, respectively. Repeated measures ANOVA summary with Tukey’s multiple comparisons test was used to compare the measurements for T Min in ROIs 6 and 9, T Aver in ROIs 2, 4, and 6–11, and T Max in ROIs 1–3, 5–9, and 11. Friedman test with Dunn’s multiple comparisons test was used to compare the measurements for LAC 0–2, T Min in ROIs 1–5, 7, 8, 10, and 11, T Aver in ROIs 1, 3, and 5, and T Max in ROIs 4 and 10, as well as to compare ROIs.

Correlations between LAC and temperature were tested independently for 0, 1, and 2 measurements. The Spearman correlation coefficient (ρ, rho) was used because of the non-Gaussianity of part of the data, especially LAC 0–2. The value of (ρ) reflected the consistency when the *p*-value was considered significant.

All results were reported as mean + SD. All statistical analyses were performed using GraphPad Prism6 software (GraphPad Software Inc., San Diego, CA, USA), where the significance level was established as *p* < 0.05.

## 3. Results

The descriptive statistics (mean ± SD) for the ROI temperatures (°C) and the blood lactate concentrations (mmol/L) are presented in Table 2. Both LAC and temperature increased immediately after exercise in comparison to the basic values before training. With time after training, all measured values decreased. After 30 min, the values of T Max in ROIs 1, 2, 4, 10, and 11, the T Min in ROIs 1–5 and 7–11, and the T Aver in ROIs 1, 2, 5, 7, and 11 returned to pre-exercise baseline, whereas values of LAC T Max in ROIs 3 and 5–9, T Min in ROI 6, and T Aver in ROIs 3, 4, 6, and 8–10 were higher than before the effort.

**Figure 3:**
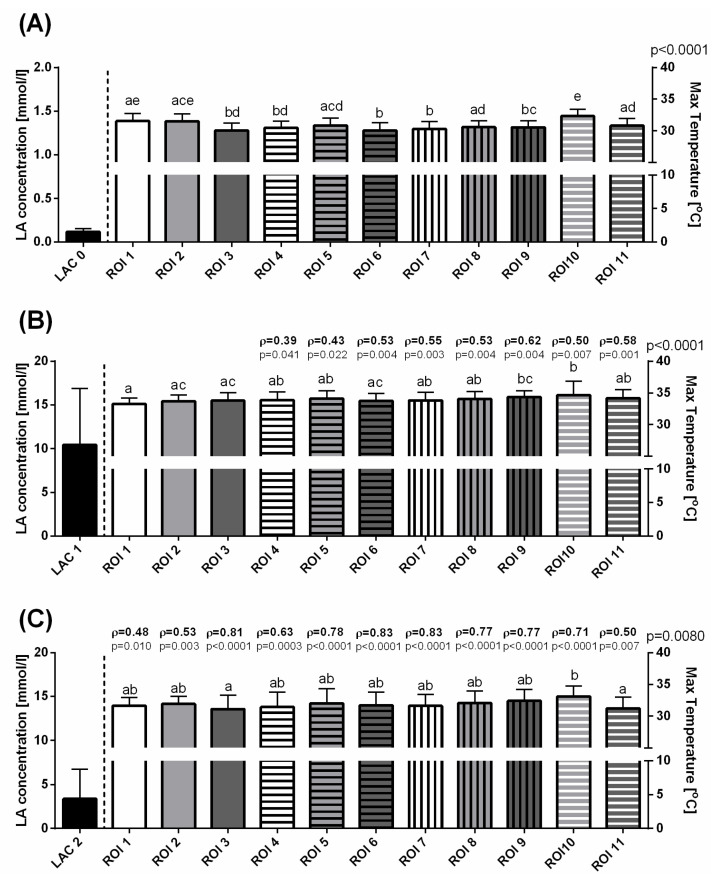
The blood lactate concentration and maximal temperatures (mean + SD) in the selected ROIs before training (**A**), immediately after exercise (**B**), and 30 min after exercise (**C**). Lowercase letters indicate differences between regions of interest (ROIs) for *p* < 0.05. The *p*-value for ROI comparison is indicated above the right *X*-axis. The correlations between blood lactate concentration and maximum temperatures are indicated above consecutive temperature bars using the Spearman correlation coefficient (ρ), which reflected the consistency when *p* < 0.05.

**Figure 4:**
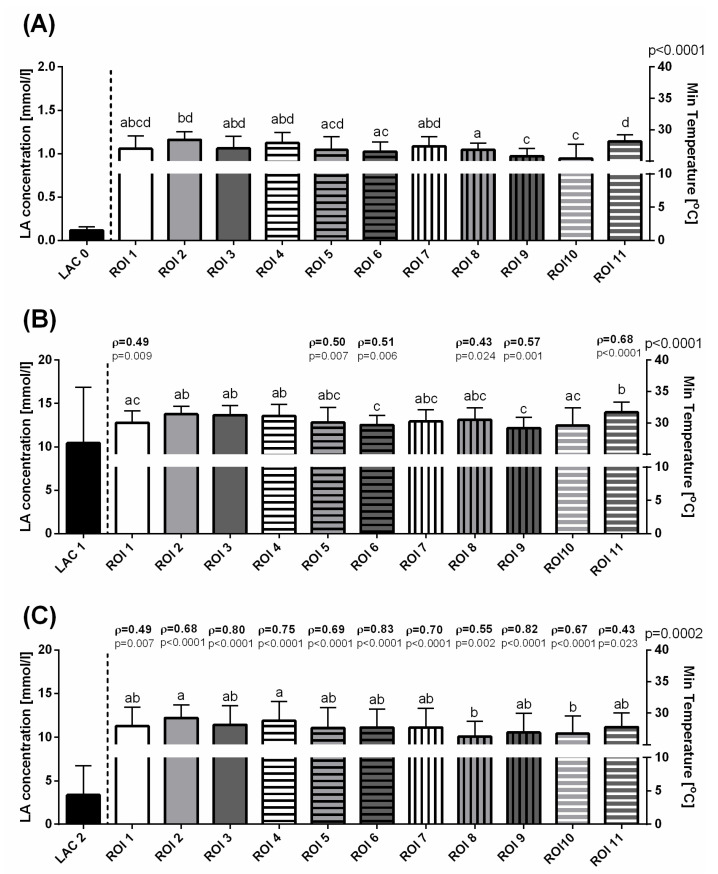
The blood lactate concentration and minimal temperatures (mean + SD) in the selected ROIs before training (**A**), immediately after exercise (**B**), and 30 min after exercise (**C**). Lowercase letters indicate differences between regions of interest (ROIs) for *p* < 0.05. The *p*-value for ROI comparison is indicated above the right *X*-axis. The correlations between blood lactate concentration and minimum temperatures are indicated above consecutive temperature bars using the Spearman correlation coefficient (ρ), which reflected the consistency when *p* < 0.05.

**Figure 5:**
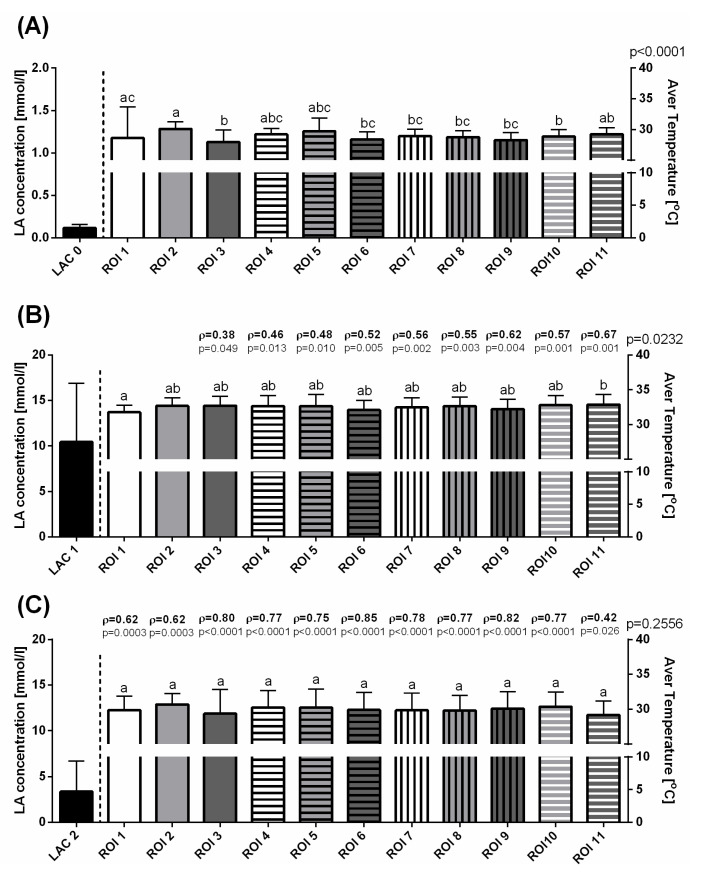
The blood lactate concentration and average temperatures (mean + SD) in the selected ROIs before training (**A**), immediately after exercise (**B**), and 30 min after exercise (**C**). Lowercase letters indicate differences between regions of interest (ROIs) for *p* < 0.05. The *p*-value for ROI comparison is indicated above the right *X*-axis. The correlations between blood lactate concentration and average temperatures are indicated above consecutive temperature bars using the Spearman correlation coefficient (ρ), which reflected the consistency when *p* < 0.05.

Before exercising, some variations in T Max (Figure 3A), T Min (Figure 4A), and T Aver (Figure 5A) were visible as significant differences between consecutive ROIs. In the first measurement, a lack of significant correlation was found between LAC and T Max, T Min, and T Aver in all ROIs (Figure 3A, Figure 4A, Figure 5A). Immediately after exercise, the T Max increased to comparable levels in ROIs 1–8 and 11, as well as significantly higher in ROIs 9 and 10 (Figure 3B). The increase in T Min values differed between ROIs in such a way that the highest T Min immediately after exercise was noted in ROI 11 and the lowest in ROIs 6 and 9 (Figure 4B). Similarly to T Max in the second measurement, T Aver increased to comparable levels in ROIs 1–10, and became significantly higher in ROIs11 (Figure 5B). In the third measurement, 30 min after exercise, a gradual decrease in values of superficial temperatures differed between ROIs for T Max and T Min. A higher T Max was noted in ROI 10 than in ROIs 3 and 11 (Figure 3C), whereas lower T Min values were noted in ROIs 8 and 10 than in ROIs 2 and 4 (Figure 4C). No differences were detected between T Aver in all ROIs (Figure 5C).

The Spearman rank correlation coefficients (ρ) between the changes in blood lactate concentration and surface temperature measures were found for the parts of ROIs immediately after exercise and for all ROIs 30 min after exercise. In the second measurement, moderately positive correlations were found between LAC and T Max changes in ROIs 4–11 (Figure 3B), LAC and T Min changes in ROIs 1, 5, 6, 8, 9, and 11 (Figure 4B), and LAC and T Aver changes in ROIs 4–11. In this period, a single weak correlation was noted for T Aver in ROI 3 (Figure 5B). In the third measurement, moderately positive correlations were found between LAC and T Max changes in ROIs 1, 2, 4, and 11 (Figure 3C), T Min changes in ROIs 1, 2, 5, 8, 10, and 11 (Figure 4C), and T Aver changes in ROIs 1, 2, and 11 (Figure 5C). Also 30 min after exercise, strong positive correlations were found for features pars LAC/T Max in ROIs 3 and 5–10 (Figure 3C), LAC/T Min in ROIs 3, 4, 6, 7, and 9 (Figure 4C), and LAC/T Aver in ROIs 3–10 (Figure 5C). The highest Spearman rank correlation coefficient values were found in ROI 6 for T Max, T Min, and T Aver.

## 4. Discussion

This was the first study confirming that the temperature of the body surface is correlated with blood LAC in race horses. Approximately 70–80% of energy produced during exercise by working muscles is released as heat [26]. In human athletes, it was postulated that thermographic diagnostics allows estimation of the working muscles’ metabolism [27]. In addition, a relationship between maximal oxygen consumption (VO_2_max) during exercise and the magnitude of body surface temperature was previously found [28]. However, in horses, only a few studies exist that evaluated IRT usefulness during training monitoring. Most of this research was performed to identify the source of lameness [29,30], as well as the influence of the rider and saddle mass on the saddle fit [31]. One publication discussed thermographic horse and rider matching during leisure riding [32]. In other studies, horse predisposition to effort was estimated [19,20]. In our study, the highest increase in T Max, T Min, and T Aver occurred in ROIs 9–11 (*m. latissimus dorsi, m. obliquus externus abdominis,* and *m. pectoralis transversus* regions) immediately after exercise, possibly as a consequence of the most loaded muscle during that type of exercise. The most recent work assessed the effects of training on hematological and biochemical blood parameters [33], as well as on the changes in body surface temperature in horses [21]. The authors indicated a significantly higher surface temperature in neck, back, gluteal, and quarter muscles after training, connected with the dynamics of changes in HCT, CPK, and, urea. However, the authors did not investigate LAC. Soroko et al. [21] used a treadmill in their study, but the biomechanical differences between exercise on a treadmill and a racetrack should be emphasized [2,34]. On the treadmill, the length of the gait is longer than for overground conditions [2]. Moreover, different stiffness levels compared to a racetrack costs the horse different amounts of energy [34]. Another issue is that, during race training, the exercise is performed with a rider.

Comparable increases of all measured superficial temperatures (T Max, T Min, and T Aver) were detected in ROIs 3, 4, and 5, which corresponded to the neck region, and ROIs 6 and 9, which corresponded to the back region. In all of these ROIs, the study demonstrated stronger correlations 30 min after exercise than immediately after. In addition, ROI 6 turned out to be most strongly associated with changes in LAC. In previous studies [21], the back region was not divided into impact areas of superficial *m. trapezius pars thoracica* (ROI 6) and *m. latissimus dorsi* (ROI 9), both of which play a role in limb retraction. These muscles support the back by pulling body of the horse forward, playing a critical role during galloping [35,36]. However, their architectural design and fiber type composition differ from each other. *M. trapezius pars thoracica* is built with a higher content of type I fibers compared to *m. latissimus dorsi* [37]. Type I fibers have relatively higher capacity for oxidative phosphorylation [38], producing a huge amount of energy by ATP synthesis and heat generation. Thus, the higher superficial temperature of *m. trapezius pars thoracica* and resulting stronger correlation with LAC than *m. latissimus dorsi* may be caused by enhanced heat production.

Based on these results, we suggest that measurements taken after 30 min provide more informative data about changes in the metabolic activity of tissues than just after exercise. In human athletes, it was documented that total body surface temperature during the initial stage of running decreases at the beginning of the work, followed by a gradual increase over time [39]. In this study the authors explained that this was a result of the vasoconstrictor and vasodilatory response, with the former lasting as long as the exercise is continued, whereas dilatation of the blood vessels starts after the end of physical activity. Vasoconstriction reduces blood flow and helps retain heat, whereas dilatation of the diameter of blood vessels leads to release of the heat [40]. In another study, it was suggested that fully meeting the metabolic and thermoregulatory demands of working tissues takes time [41]. Thus, thermography measurements used to evaluate the efficiency of recovery can be considered to be the most valid.

The main limitation of our study was the number of race horses in the examined group, which was too small to attempt to establish the reference thermographic values. In addition, only limited parts of the body were investigated in the study. Further studies should also take into account the proximal hind limb region (e.g., *m. gluteus*, *m. semitendinosus*, *m. semimembranosus*, *quadriceps,* and *biceps femoris*), which may also be indicated as interesting in the monitoring of metabolic changes in horses after training [21]. IRT and blood collection at the same time is a time-consuming process, so we tried not to disturb the trainers’ and jockeys’ work. It should also be emphasized that the handheld lactate Accusport^®^ analyzer was used in this study, which is regarded as reliable tool in measuring LAC in horses comparatively with other analyzers [42,43]. However, differences between blood LAC blood values are still likely to exist due to variation in laboratory evaluation techniques.

## 5. Conclusions

This study fills the existing gap in the literature about equine exercise physiology. Evaluation of the blood LAC changes in connection with IRT provided more information during monitoring of the classic race training process, suggesting that the *musculus trapezius pars thoracica* region 30 min after training is the most suitable for postexercise temperature evaluation. The monitoring of training progress is particularly important to assess optimal training and the best sports results in race horses. As a noninvasive procedure, IRT may supplement widely accepted hematological and biochemical measurements to evaluate adaptation to increased workload during race training. However, more studies are necessary to estimate appropriate reference values.

## Figures and Tables

**Table 1:** The characteristics of the regions of interest (ROIs) taken into consideration during the body surface temperature evaluation.

No.	Areas with Impact of Superficial Tissues	Range of ROI
ROI 1	Arteries in the Viborg’s triangle	A line from the lateral surface of the atlas to the ventral part of the angle of mandible
ROI 2	vena jugular externa	A line along the sulcus jugularis
ROI 3	M. brachiocephalicus	A parallelogram-shaped area from the lateral surface of the atlas, behind the angle of the mandible, to the regio supraspinata of the scapula
ROI 4	Mm. splenius capitis and cervicis	A triangle-shaped area from the lateral surface of the axis to the regio supraspinata of the scapula above ROI 3
ROI 5	M. trapezius pars cervicalis	A triangle ranging from the middle of the neck to the regio cartilaginis of the scapula and along the regio supraspinata of the scapula, up to two-thirds of the length
ROI 6	M. trapezius pars thoracica	A triangle ranging from the the regio cartilaginis of the scapula along the regio supraspinata of the scapula, up to one-third of the length
ROI 7	M. deltoideus	An irregular area in the projection of the regio supraspinata of the scapula
ROI 8	M. pectoralis descendens	An irregular area in the projection of the regio infraspinata of the scapula
ROI 9	M. latissimus dorsi	A triangle-shaped area from the regio infraspinata of the scapula, up to two-thirds of the length, along the back to the tuber coxae
ROI 10	M. obliquus externus abdominis	A trapezoid-shaped area from the lower two-thirds of the regio infraspinata of the scapula to the tuber coxae and the region of the processus xiphoideus sterni
ROI 11	M. pectoralis transversus	A triangle-shaped area behind the region of the olecranon to the region of the processus xiphoideus sterni

ROI, region of interest; m., muscle.

**Table 2:** The blood lactate concentration and temperature measurements (mean ± SD) in the selected ROIs before training (0, *n* = 40), immediately after exercise (1, *n* = 40), and 30 min after exercise (2, *n* = 40).

Measurement		0	−1	−2	*p*-Value
LAC (mmol/L)		0.12 ± 0.04 ^a^	10.43 ± 6.44 ^b^	3.37 ± 3.35 ^c^	*p* < 0.0001
ROI 1	T Max	31.53 ± 1.22 ^a^	33.23 ± 0.99 ^b^	31.62 ± 1.29 ^a^	*p* < 0.0001
	T Min	26.98 ± 2.08 ^a^	30.04 ± 1.87 ^b^	27.92 ± 3.01 ^a^	*p* < 0.0001
	T Aver	28.64 ± 5.06 ^a^	31.76 ± 1.03 ^b^	29.84 ± 2.05 ^a^	*p* < 0.0001
ROI 2	T Max	31.49 ± 1.20 ^a^	33.69 ± 0.99 ^b^	31.89 ± 1.25 ^a^	*p* < 0.0001
	T Min	28.40 ± 1.27 ^a^	31.39 ± 1.26 ^b^	29.23 ± 2.09 ^a^	*p* < 0.0001
	T Aver	30.10 ± 1.16 ^a^	32.69 ± 1.13 ^b^	30.68 ± 1.58 ^a^	*p* < 0.0001
ROI 3	T Max	30.06 ± 1.15 ^a^	33.79 ± 1.22 ^b^	31.10 ± 2.22 ^c^	*p* < 0.0001
	T Min	27.04 ± 1.93 ^a^	31.25 ± 1.53 ^b^	28.14 ± 3.04 ^a^	*p* < 0.0001
	T Aver	27.99 ± 1.95 ^a^	32.71 ± 1.36 ^b^	29.37 ± 3.44 ^c^	*p* < 0.0001
ROI 4	T Max	30.44 ± 1.06 ^a^	33.86 ± 1.28 ^b^	31.43 ± 2.35 ^a^	*p* < 0.0001
	T Min	27.92 ± 1.63 ^a^	31.10 ± 1.86 ^b^	28.82 ± 3.00 ^a^	*p* < 0.0001
	T Aver	29.22 ± 0.96 ^a^	32.66 ± 1.53 ^b^	30.23 ± 2.45 ^c^	*p* < 0.0001
ROI 5	T Max	30.85 ± 1.15 ^a^	34.09 ± 1.27 ^b^	32.01 ± 2.30 ^a^	*p* < 0.0001
	T Min	26.80 ± 2.11 ^a^	30.11 ± 2.36 ^b^	27.65 ± 3.24 ^a^	*p* < 0.0001
	T Aver	29.73 ± 2.15 ^a^	32.63 ± 1.70 ^b^	30.23 ± 2.67 ^a^	*p* < 0.0001
ROI 6	T Max	30.06 ± 1.20 ^a^	33.76 ± 1.17 ^b^	31.68 ± 2.11 ^c^	*p* < 0.0001
	T Min	26.53 ± 1.55 ^a^	29.68 ± 1.50 ^b^	27.73 ± 2.91 ^c^	*p* < 0.0001
	T Aver	28.39 ± 1.22 ^a^	32.10 ± 1.39 ^b^	29.89 ± 2.54 ^c^	*p* < 0.0001
ROI 7	T Max	30.29 ± 1.16 ^a^	33.83 ± 1.26 ^b^	31.60 ± 1.82 ^c^	*p* < 0.0001
	T Min	27.35 ± 1.54 ^a^	30.25 ± 1.84 ^b^	27.72 ± 3.02 ^a^	*p* < 0.0001
	T Aver	28.95 ± 1.10 ^a^	32.48 ± 1.38 ^b^	29.85 ± 2.46 ^a^	*p* < 0.0001
ROI 8	T Max	30.59 ± 1.02 ^a^	34.05 ± 1.20 ^b^	32.06 ± 1.87 ^c^	*p* < 0.0001
	T Min	26.83 ± 1.06 ^a^	30.51 ± 1.88 ^b^	26.31 ± 2.41 ^a^	*p* < 0.0001
	T Aver	28.77 ± 1.03 ^a^	32.63 ± 1.33 ^b^	29.83 ± 2.16 ^c^	*p* < 0.0001
ROI 9	T Max	30.54 ± 1.04 ^a^	34.33 ± 1.03 ^b^	32.39 ± 1.81 ^c^	*p* < 0.0001
	T Min	25.79 ± 1.26 ^a^	29.20 ± 1.67 ^b^	26.95 ± 3.03 ^a^	*p* < 0.0001
	T Aver	28.30 ± 1.21 ^a^	32.22 ± 1.43 ^b^	30.08 ± 2.42 ^c^	*p* < 0.0001
ROI 10	T Max	32.32 ± 1.08 ^a^	34.67 ± 2.20 ^b^	33.10 ± 1.61 ^a^	*p* < 0.0001
	T Min	25.43 ± 2.24 ^a^	29.58 ± 2.83 ^b^	26.79 ± 2.79 ^a^	*p* < 0.0001
	T Aver	28.87 ± 1.14 ^a^	32.79 ± 1.40 ^b^	30.37 ± 2.09 ^c^	*p* < 0.0001
ROI 11	T Max	30.80 ± 1.13 ^a^	34.18 ± 1.32 ^b^	31.20 ± 1.80 ^a^	*p* < 0.0001
	T Min	28.14 ± 1.10 ^a^	31.69 ± 1.63 ^b^	27.78 ± 2.23 ^a^	*p* < 0.0001
	T Aver	29.27 ± 1.04 ^a^	32.87 ± 1.44 ^b^	29.19 ± 2.00 ^a^	*p* < 0.0001

LAC, blood lactate concentration; T Max, maximal temperature; T Min, minimal temperature; T Aver, average temperature; ROI, regions of interest. Differences between measurements (0, 1, 2) are indicated by a, b, and c. The significance level was established as *p* < 0.05.
